# Study of Triangular Fuzzy Hybrid Nanofluids on the Natural Convection Flow and Heat Transfer between Two Vertical Plates

**DOI:** 10.1155/2021/3678335

**Published:** 2021-11-11

**Authors:** Muhammad Nadeem, Ahmed Elmoasry, Imran Siddique, Fahd Jarad, Rana Muhammad Zulqarnain, Jawdat Alebraheem, Naseer S. Elazab

**Affiliations:** ^1^Department of Mathematics, University of Management and Technology, Lahore 54770, Pakistan; ^2^Department of Mathematics, College of Science Al Zufli, Majmaah University, Majmaah 11952, Saudi Arabia; ^3^Department of Mathematics, Cankaya University, Etimesgut, Ankara, Turkey; ^4^Department of Medical Research, China Medical University Hospital, China Medical University, Taichung, Taiwan; ^5^Department of Mathematics, University of Management and Technology, Lahore, Sialkot Campus, Pakistan; ^6^Department of Mathematics, Faculty of Science, Cario University, Giza, Egypt

## Abstract

The prime objective of the current study is to examine the effects of third-grade hybrid nanofluid with natural convection utilizing the ferro-particle (Fe_3_O_4_) and titanium dioxide (TiO_2_) and sodium alginate (SA) as a host fluid, flowing through vertical parallel plates, under the fuzzy atmosphere. The dimensionless highly nonlinear coupled ordinary differential equations are computed adopting the bvp4c numerical approach. This is an extremely effective technique with a low computational cost. For validation, it is found that as the volume fraction of (Fe_3_O_4_+TiO_2_) hybrid nanoparticles rises, so does the heat transfer rate. The current and existing results with their comparisons are shown in the form of the tables. The present findings are in good agreement with their previous numerical and analytical results in a crisp atmosphere. The nanoparticles volume fraction of Fe_3_O_4_ and TiO_2_ is taken as uncertain parameters in terms of triangular fuzzy numbers (TFNs) [0, 0.05, 0.1]. The TFNs are controlled by *α* − cut and the variability of the uncertainty is studied through triangular membership function (MF).

## 1. Introduction

Researchers have been attracted by natural convection (NC) flow because of its numerous uses in engineering and scientific problems like heat exchangers, building ventilation, insulation, solar energy collection, refrigeration, nuclear waste repositories, petroleum reservoirs geothermal systems, and chemical catalytic reactors. Convection is used significantly in the manufacturing of solar panels, microstructures during the cooling of molten metals, and free air cooling without the need for fans in real-world applications. Various researchers have looked into the NC-based flow of non-Newtonian and Newtonian fluids between two infinite parallel vertical plates such as Bruce and Na [1] who investigated the heat transfer of NC between vertical flat plates using non-Newtonian Powell–Eyring fluids. Later on, Rajagopal and Na [2] studied the extensive thermodynamic analysis on fundamental functions. The influences of the third-grade non-Newtonian fluid on heat transfer (HT) were examined by Ziabakhsh and Domairry [3] through the homotopy analysis method (HAM). Using the least square method (LSM), Maghsoudi et al. [4] inspected the NC flow of third-grade fluid between two infinite vertical flat plates with a porous media. Mansoor et al. [5] studied the natural convective flow between two vertical plates with the help of the volume of parameter method (VPM) and Runge–Kutta method (RKM). They show that VPM is better than RKM. Some researchers have explored different flows of fluids between vertical parallel plates analytically and numerically [6, 7].

Because of the escalation in energy prices, HT management is extremely important in energy systems. So the nanofluids are the mixtures of liquid and nanoparticles which are used to improve the rate of heat transfer. The performance of nanoparticles in a heat transfer mechanism is excellent as compared to regular fluid. This is due to the dispersed ultrafine particles boosting the thermal conductivity of the fluid and therefore increasing their energy transfer competency. In this way, convective HT of nanofluid is a challenging problem. When a non-Newtonian fluid is moving in a structure, natural convection analysis is a difficult task. The nanoparticle adds to the base fluid and then heat transfer may be increased and this added nanoparticle in the base fluid is named nanofluid [8, 9]. Numerous literatures [10, 11] disclose the low volume fractions (1–4 volume %) for better performance of thermal conductivity of the fluids. We can utilise nanoparticle concentrations of greater than 20% [12]. When two or more distinct nanoparticles are added to the base fluid, it is referred to as “hybrid nanofluid,” also the thermal conductivity is greater than as compared to nanofluid and regular fluid. In the field of heat transfer, hybrid nanofluids have received a lot of attention such as nuclear system cooling, drug reduction, automobile radiators, thermal storage, welding, electronic cooling, solar heating, lubrication, the coolant in machining, generator cooling, defence, biomedical, heating, and refrigeration, etc. Saqib et al. [13] studied the NC flow problem on Jaffry hybrid nanofluid using CNTs (single- and multiwall carbon nanotubes) with carboxy-methyl-cellulose (CMS) as a base fluid between two vertical parallel plates. Hatami and Ganji [14] applied the differential transform method (DTM) to investigate the NC flow of sodium alginate (SA) as a host fluid and silver (Ag) and copper (Cu) as nanofluids between two vertical parallel plates. Maghsoudi et al. [15] investigated natural convective, thermal radiation, HT, and magnetic field of the non-Newtonian nanofluid flow between two infinite vertical flat plates utilizing the Galerkin method (GM). Using the HAM, Rahmani et al. [16] explored the NC flow of non-Newtonian nanofluids between two vertical plates. They observed that HAM is better than the numerical RK method. The NC flow of non-Newtonian nanofluids between two vertical plates using the generalized decomposition method (GDM) was also studied by Kezzar et al. [17]. They observed that GDM is better than the numerical RK method. Biswal et al. [18, 19] used the HPM in an uncertain environment to examine the NC of nanofluid flow between two parallel plates. The volume fraction of nanoparticle was considered as TFN and also shows the fuzzy result is better than a crisp result. Gabli et al. [20] studied the NC flow of non-Newtonian ferro-particle (Fe_3_O_4_) nanofluids between two vertical plates with thermal radiation using the Adomian decomposition method (ADM). They observed that ADM is better than the RK-Feldberg-based shooting method. Devi and Devi [21] inspected the HT and flow problems of hydro-magnetic hybrid nanofluids (Al_2_O_3_+(Cu/H_2_O)) through a stretched sheet.

Fluid flow with heat transfer is essential in science and engineering. Because of extensive physical properties such as chemical diffusion, magnetic effect, and heat transfer, governing fluid equations are converted into linear or nonlinear DEs. After controlling these physical issues, they are transformed into linear or nonlinear DEs. The solution of DEs is strongly affected by the physical problems with associated parameters and initial, geometry, coefficient, and boundary conditions. Then, these are not crisp due to the mechanical defect, experimental error, and measurement error, etc. In this scenario, fuzzy sets theory (FST) is a more accurate instrument than assuming genuine physical problems for getting a better understanding of the facts under investigation. To be more specific, FDEs are useful for decreasing uncertainty and determining the best way to define a physical problem with unknown parameters and initial and boundary conditions.

The FST was first presented by Zadeh [21] in 1965. FST is a fantastic approach for describing circumstances when information is unclear, imprecise, or uncertain. Later on, Dubois and Prade [22] developed arithmetic procedures on fuzzy numbers (FNs). The trapezoidal, triangular, and Gaussian FNs are three forms of FNs that may be classified. For thoroughness, we will look at TFNs now. The FN is a variable that has a range from 0 to 1. Each numerical value in the range is given a membership grade, with 0 being the lowest grade and 1 being the strongest possible grade. The information contained in crisp partial or ordinary differential equations models of dynamical systems is sometimes incomplete, imprecise, or ambiguous. FDEs are a useful approach for modelling dynamical systems with ambiguity or uncertainty. FNs or TFNs can be used to define this impreciseness or vagueness mathematically. Many researches have been conducted in recent years around the notion of FDEs. The fuzzy differentiability idea was established by Seikala [23] and Kaleva [24] and then they discussed fuzzy integration and differentiation. The FDEs were first presented in 1987 by Kandel and Byatt [25]. For the solution of FDEs, Buckley et al. [26] employed two methods: the extension principle and FNs. For continuous FDEs, Nieto [27] investigated the Cauchy problem. In [28], Lakshmikantham and Mohapatra investigated the initial value problems for FDEs. For the existence and uniqueness solution of FDE, Park and Hyo [29] employed the successive approximation approach. The geometric approach for solving a system of FDEs was devised by Gasilov et al. [30]. The system of FDEs with TFNs was investigated by Nizami et al. [31]. Salahsour et al. [32] used FDE and TFNs to investigate the fuzzy alley effect and the fuzzy logistic equation.

In addition, numerous scholars have used FST to achieve well-known findings in commerce and science, for example, in bank account model [33], population dynamics model [34, 35], bacteria culture model [36], HIV model [37], growth model [38], computational biology [39], modelling hydraulic [40], predator-prey model [41], quantum optics and gravity [42], decay model [43], model of friction [44], civil engineering [45], Laplace transform [46], integro-differential equation [47], dengue virus model [48], chemostat model [49], and giving up smoking model [50]. The adaptive fuzzy controller for uncertain fractional-order nonlinear systems was proposed by Liu et al. [51], and it was used to evaluate an unknown nonlinear function. Very recently, Nadeem et al. [52] recently investigated MHD and ohmic heating on the third-grade fluid in an inclined channel under the fuzzy environment. The triangle membership function was used to discuss the uncertainty.

Inspired by the earlier investigations, the goal of this paper is to use the numerical scheme bvp4c to analyze the hybrid nanofluid flow between two vertical parallel plates in a fuzzy environment. The sodium alginate (SA) is the host fluid, while the hybrid nanoparticles are Fe_3_O_4_ and TiO_2_. The impacts of the Eckert number, Prandtl number, and nanoparticle volume friction on velocity and temperature profiles are studied. It has been detected that hybrid nanofluid enhanced the thermal efficiency of the base fluid rapidly as compared to the other fluid and nanofluids. Besides, after checking the accuracy of bvp4c so compare the results of existing work in the literature. The nanoparticle volume fraction has also been treated as an uncertain parameter in this investigation, with fuzzy numbers or triangular fuzzy numbers being used. The FDEs with *α* − cut approach are used to tackle the natural convection problem in a fuzzy environment.

The paper is arranged as follows. We discussed some fundamental preliminaries on FDEs in Section 2. The formation of the crisp problem is described in Section 3. The crisp problem is transformed into FDEs in Section 4.  Section 5 contains an explanation of graphs and tables.  Section 6 concludes with some closing remarks.

## 2. Preliminaries

Some fundamental definitions are given in this section.

**Definition 1 (see [21, 52]).**

Fuzzy set is defined as a set of ordered pairs such that $\tilde{U}=\left\{\left(y,\mu_{\tilde{U}}\left(y\right)\right):\;y\in X,\mu_{\tilde{U}}\left(y\right)\in \left[0,1\right]\right\}$, where $\mu_{\tilde{U}}\left(y\right)$ is the membership function of $\tilde{U}$, *X* is the universal set, and mapping is defined as $\mu_{\tilde{U}}\left(y\right):X⟶\left[0,1\right]$.

**Definition 2 (see [21, 52]).**

*α*-cut or *α*-level of a fuzzy set $\tilde{U}$ is a crisp set *U*_*α*_ and defined by $U_{\alpha }=\left\{\left(y/\mu_{\tilde{U}}\left(y\right)\right)\geq \alpha \right\}$, where 0 ≤ *α* ≤ 1.

**Definition 3 (see [22, 52]).**

Let $\tilde{U}=\left(a_{1},a_{2},a_{3}\right)$ with membership function $\mu_{\tilde{U}}\left(y\right)$ which is called a TFN if

(1) $$\begin{array}{c} \mu_{\tilde{U}}\left(y\right)=\left\{\begin{array}{cc} \frac{a_{1}-y}{a_{2}-a_{1}}, & \text{for }y\in \left[a_{1},a_{2}\right],\\ \frac{y-a_{3}}{a_{2}-a_{3}}, & \text{for }y\in \left[a_{2},a_{3}\right],\\ 0, & \text{otherwise}. \end{array}\right. \end{array}$$

**Figure 1:**
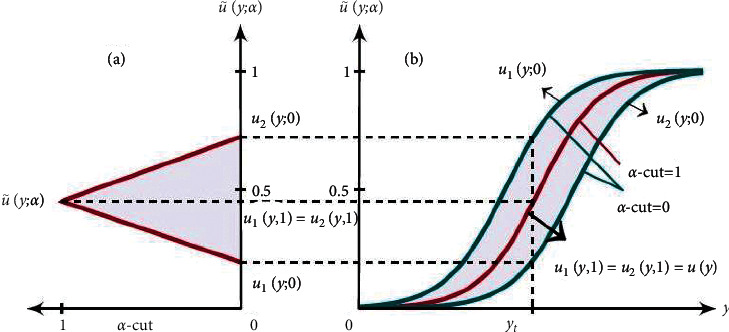
Membership functions of a TFN.

The TFN with peak (or center) *a*_2_, left width *a*_2_ − *a*_1_ > 0, right width *a*_3_ − *a*_2_ > 0, and these TFNs are transformed into interval numbers through *α*-cut approach, is written as $\tilde{U}=\left[u_{1}\left(y;\alpha \right),u_{2}\left(y;\alpha \right)\right]=\left[a_{1}+\left(-a_{1}+a_{2}\right)\alpha ,a_{3}-\left(-a_{2}+a_{3}\right)\alpha \right]$, where *α* ∈ [0,1]. The membership function is the building block of FST and it is defined by its membership function. TFNs $\tilde{U}=\left(a_{1},a_{2},a_{3}\right)$ and *α*-cut of membership function are shown in Figure 1. An arbitrary TFN satisfies the following conditions: (i) *u*_1_(*y*; *α*) is an increasing function on [0, 1]. (ii) *u*_2_(*y*; *α*) is a decreasing function on [0, 1]. (iii) *u*_1_(*y*; *α*) ≤ *u*_2_(*y*; *α*) on [0, 1]. (iv) *u*_1_(*y*; *α*) and *u*_2_(*y*; *α*) are bounded at [0, 1], respectively. (v) If *u*_1_(*y*, *α*)=*u*_2_(*y*, *α*)=*u*(*y*) where *u*(*y*) is a crisp number.

**Definition 4 (see [23, 25, 52]).**

Let I be a real interval. A mapping $\tilde{u}\left(y;\alpha \right):I⟶F$ is called a fuzzy process, defined as $\tilde{u}\left(y;\alpha \right)=\left[u_{1}\left(y;\alpha \right),u_{2}\left(y;\alpha \right)\right],\;y\in I$ and *α* ∈ [0,1]. The derivative $\left(\text{d}\tilde{u}\left(y;\alpha \right)/\text{d}y\right)\in F$ of a fuzzy process $\tilde{u}\left(y;\alpha \right)$ is defined by $\left(\text{d}\tilde{u}\left(y;\alpha \right)/\text{d}y\right)=\left[\left(\text{d}u_{1}\left(y;\alpha \right)/\text{d}y\right),\left(\text{d}u_{2}\left(y;\alpha \right)/\text{d}y\right)\right]$.

**Definition 5 (see [23, 25, 52]).**

Let $I\subseteq R,\tilde{u}\left(y;\alpha \right)$ be a fuzzy valued function defined on *I*. Let $\tilde{u}\left(y;\alpha \right)=\left[u_{1}\left(y;\alpha \right),u_{2}\left(y;\alpha \right)\right]$ for all *α*-cut. Assume that *u*_1_(*y*; *α*) and *u*_2_(*y*; *α*) have continuous derivatives or are differentiable, for all *y* ∈ *I* and *α*, then $\left(\text{d}\tilde{u}\left(y;\alpha \right)/\text{d}y\right)={\left[\left(\text{d}u_{1}\left(y;\alpha \right)/\text{d}y\right),\left(\text{d}u_{2}\left(y;\alpha \right)/\text{d}y\right)\right]}_{\alpha }$. Similarly, we can define higher-order ordinary derivatives in the same way. An FN by an ordered pair of functions ${\left[\text{d}\tilde{u}\left(y;\alpha \right)/\text{d}y\right]}_{\alpha }$ and they satisfy the following conditions: (i) (d*u*_1_(*y*; *α*)/d*y*) and (d*u*_2_(*y*; *α*)/d*y*) are continuous on [0, 1]. (ii) (d*u*_1_(*y*; *α*)/d*y*) is an increasing function on [0, 1]. (iii) (d*u*_2_(*y*; *α*)/d*y*)is a decreasing function on [0, 1]. (iv) (d*u*_1_(*y*; *α*)/d*y*) ≤ (d*u*_2_(*y*; *α*)/d*y*) on [0, 1].

## 3. Problem Formulation

**Figure 2:**
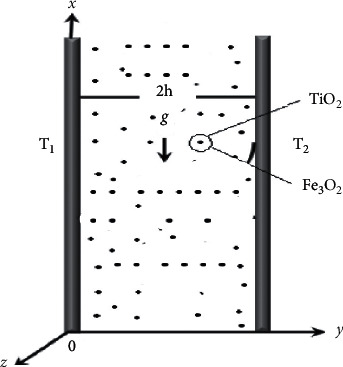
Flow geometry.

In this proposed problem, Figure 2 portrays the main theme schematically. It consists of two vertical parallel flat plates separated by a distance 2*h* apart, in which there is a non-Newtonian fluid, which is flowing due to the free convection. The walls at *x* *=* *h* and *x* *=* −*h* are held at constant temperatures *T*_1_ and *T*_2_, respectively, with (*T*_1_ > *T*_2_). This difference of temperature causes the fluid near the walls at *x* *=* −*h* to rise and the fluid near the wall *x*=*b* to fall. The fluid is a non-Newtonian sodium alginate-based nanofluid containing Fe_3_O_4_ and TiO_2_ hybrid nanoparticles. The base fluid and the hybrid nanoparticles are considered to be in thermal equilibrium, with no-slip between them. Some physical properties of the hybrid nanofluid are arranged in Table 1.

Using the above assumptions and Boussinesq approximation [14], the momentum and energy equations of the natural convection flow of an incompressible third-grade nanofluid are as follows [2, 3, 5, 14, 20].

The equation of motion is

(2) $$\begin{array}{c} \mu_{hnf}\frac{{\text{d}}^{2}u}{\text{d}y^{2}}+6\beta_{3}{\left(\frac{\text{d}u}{\text{d}y}\right)}^{2}\frac{{\text{d}}^{2}u}{\text{d}y^{2}}+{\left(\beta_{T}\rho \right)}_{hnf}\left(T-T_{m}\right)g=0, \end{array}$$

and the equation of energy is as follow:

(3) $$\begin{array}{c} K_{hnf}\frac{{\text{d}}^{2}T}{\text{d}y^{2}}+\mu_{hnf}{\left(\frac{\text{d}V}{\text{d}y}\right)}^{2}+2\beta_{3}{\left(\frac{\text{d}V}{\text{d}y}\right)}^{4}=0, \end{array}$$

with the following boundary conditions:

(4) $$\begin{array}{c} u\left(y\right)=0,\\ \theta \left(y\right)=T_{1},\;\text{at }y=-h,\\ u\left(y\right)=0,\\ \theta \left(y\right)=T_{2}\;\text{at }y=-h. \end{array}$$

The dimensionless variables [2]

(5) $$\begin{array}{c} u=\frac{\bar{u}}{V_{0}},\\ y=\frac{\bar{y}}{h},\\ \theta =\frac{T-T_{m}}{T_{1}-T_{2}}, \end{array}$$

After removing the bar, we have

(6) $$\begin{array}{c} \frac{{\text{d}}^{2}u}{\text{d}y^{2}}+\frac{6\beta }{A_{2}}{\left(\frac{\text{d}u}{\text{d}y}\right)}^{2}\frac{{\text{d}}^{2}u}{\text{d}y^{2}}+\frac{A_{3}A_{1}\theta \text{Gr}}{A_{2}}=0, \end{array}$$

(7) $$\begin{array}{c} \frac{{\text{d}}^{2}\theta }{\text{d}y^{2}}+\frac{\text{Pr}Ec}{A}{\left(1-\phi_{1}\right)}^{2.5}{\left(1-\phi_{2}\right)}^{2.5}{\left(\frac{\text{d}u}{\text{d}y}\right)}^{2}+\frac{2\beta \text{PrEc}}{A}{\left(\frac{\text{d}u}{\text{d}y}\right)}^{4}=0, \end{array}$$

And dynamic and thermal boundary conditions are

(8) $$\begin{array}{c} u\left(y\right)=0,\\ \theta \left(y\right)=-0.5,\;\text{at }=-1,\\ u\left(y\right)=0,\\ \theta \left(y\right)=0.5,\;\text{at }y=1. \end{array}$$

(9) $$\begin{array}{c} \text{Pr}=\frac{\mu_{f}{\left(\rho C_{p}\right)}_{f}}{\rho_{f}k_{f}},\\ \text{Ec}=\frac{V_{0}^{2}\rho_{f}}{\left(T_{1}-T_{2}\right){\left(\rho C_{p}\right)}_{f}},\\ \beta =\frac{6V_{0}^{2}\beta_{3}}{h^{2}\mu_{f}},\\ \text{Gr}=\frac{\left(T_{w}-T_{\infty }\right)g{\left(\rho \beta_{T}\right)}_{f}}{h^{2}}, \end{array}$$

where the dimensionless Grashof number (Gr), the Eckert number (Ec), Prandtl number (Pr), and the non-Newtonian viscosity (*β*).

(10) $$\begin{array}{c} A_{1}=\frac{\rho_{hnf}}{\rho_{f}}=\left[\left(-\phi_{2}+1\right)\left\{\left(1-\phi_{1}\right)+\frac{\rho_{s_{1}}}{\rho_{f}}\phi_{1}\right\}+\phi_{2}\frac{\rho_{s_{2}}}{\rho_{f}}\right],\\ A_{2}=\mu_{hnf}=\frac{\mu_{f}}{{\left(1-\phi_{1}\right)}^{2.5}{\left(1-\phi_{2}\right)}^{2.5}},\\ A_{3}=\frac{{\left(\beta_{T}\right)}_{hnf}}{{\left(\beta_{T}\right)}_{f}}=\phi_{2}\frac{{\left(\beta_{T}\right)}_{s_{2}}}{{\left(\beta_{T}\right)}_{f}}+\left[\left(1-\phi_{1}\right)+\phi_{1}\frac{{\left(\beta_{T}\right)}_{s_{1}}}{{\left(\beta_{T}\right)}_{f}}\right]\left(1-\phi_{2}\right),\\ A=\frac{k_{hnf}}{k_{nf}}=\frac{2k_{nf}+2\phi_{1}\left(k_{s_{1}}-k_{nf}\right)+k_{s_{1}}}{2k_{nf}-\phi_{1}\left(k_{s_{1}}-k_{nf}\right)+k_{s_{1}}},\\ \frac{k_{nf}}{k_{f}}=\frac{2k_{f}+2\phi_{2}\left(k_{s_{2}}-k_{f}\right)+k_{s_{2}}}{2k_{f}-\phi_{2}\left(k_{s_{2}}-k_{f}\right)+k_{s_{2}}}, \end{array}$$

where *ρ*_*hnf*_, *k*_*hnf*_, *μ*_*hnf*_, (*β*_*T*_)_*hnf*_, (*ρC*_*p*_)_*hnf*_,  *ϕ*_1_,  and *ϕ*_2_ denote the density, thermal conductivity, viscosity, thermal expansion coefficient, specific heat, Fe_3_O_4_ nanoparticles volume fraction, and TiO_2_ nanoparticles volume fraction of hybrid nanofluids, respectively. [53].

## 4. Formulation of the Crisp Problem into a Fuzzy Problem Using FDEs

The velocity and temperature of nanoparticles are affected by small changes in their volume fraction. Some researchers take the nanoparticles volume fraction in this range [0.01–0.04], implying that fluid flow is solely dependent on these values. Then, due to the fixed crisp values of the volume fraction of nanoparticles, uncertainty develops.

Since *ϕ*_1_ representing the volume fraction of Fe_3_O_4_ and *ϕ*_2_ represents the volume fraction of TiO_2_, so, in a fuzzy environment, it is preferable to address a complex situation by accepting both volume fractions as FN.

For fuzzy solutions, equations (6)–(8) can be converted into FDE using *α* − cut approach. So, according to Definitions 4 and 5, we have

(11) $$\begin{array}{c} \frac{{\text{d}}^{2}}{\text{d}y^{2}}\left[u_{1}\left(y,\alpha \right),u_{2}\left(y,\alpha \right)\right]+\frac{6\beta }{A_{2}}\frac{{\text{d}}^{2}}{\text{d}y^{2}}\left[u_{1}\left(y,\alpha \right),u_{2}\left(y,\alpha \right)\right]{\left(\frac{\text{d}}{\text{d}y}\left[u_{1}\left(y,\alpha \right),u_{2}\left(y,\alpha \right)\right]\right)}^{2}\\ +\frac{A_{1}A_{3}\text{Gr}}{A_{2}}\left[\theta_{1}\left(y,\alpha \right),\theta_{2}\left(y,\alpha \right)\right]=0, \end{array}$$

(12) $$\begin{array}{c} \frac{{\text{d}}^{2}}{\text{d}y^{2}}\left[\theta_{1}\left(y,\alpha \right),\theta_{2}\left(y,\alpha \right)\right]+\frac{\text{PrEc}}{A}{\left(1-\phi_{1}\right)}^{2.5}{\left(1-\phi_{2}\right)}^{2.5}{\left(\frac{\text{d}}{\text{d}y}\left[u_{1}\left(y,\alpha \right),u_{2}\left(y,\alpha \right)\right]\right)}^{2}\\ +\frac{2\beta \text{PrEc}}{A}{\left(\frac{\text{d}}{\text{d}y}\left[u_{1}\left(y,\alpha \right),u_{2}\left(y,\alpha \right)\right]\right)}^{4}=0,\\ \theta \left(y,\alpha \right)=-0.5,\;\text{at }y=-1,\\ u\left(y,\alpha \right)=0,\\ \theta \left(y,\alpha \right)=0.5,\;\text{at }y=-1, \end{array}$$

(13) $$\begin{array}{c} u\left(y,\alpha \right)=0,\\ \theta \left(y,\alpha \right)=-0.5,\;\text{at }y=-1,\\ u\left(y,\alpha \right)=0,\\ \theta \left(y,\alpha \right)=0.5,\;\text{at }y=-1, \end{array}$$

where 0 ≤ *α* ≤ 1*u*_1_(*y*, *α*) is the lower bound and *u*_2_(*y*, *α*) is the upper bound of fuzzy velocity profiles. Similarly, the fuzzy temperature profiles are $\bar{\theta }\left(y,\alpha \right)=\left[\theta_{1}\left(y,\alpha \right),\theta_{2}\left(y,\alpha \right)\right],\;0\leq \alpha \leq 1$.

Table 2 presents the crisp values and TFNs of these FNs. The TFN defined the variation of FN at each *α* − cut.The TFNs are used to define the triangular MFs of the FNs which is ranging from 0 to 1, see Figure 1. This investigated range is commonly used to develop the aforementioned problem.

Now, we present a boundary value problem solver numerical procedure for controlling crisp differential equations (equations (6)–(8)) and FDEs (equations (11)–(13)) with boundary conditions, which are called bvp4c. It is a Lobatto IIIa formula with three stages based on the finite-difference algorithm. It has a collocation polynomial, and in [*a*, *b*], the collocation formula yields a sixth-order accurate uniform C1 continuous solution. For error control and mesh selection, the continuous solutions residual is employed. The aforementioned ODEs are transformed into a first-order system as follows:

Let

(14) $$\begin{array}{c} u\left(y\right)=m\left(1\right),\\ u^{\prime \prime }\left(y\right)=m^{\prime }\left(2\right), \end{array}$$

(15) $$\begin{array}{c} m^{\prime }\left(2\right)=\frac{-A_{1}A_{3}m\left(3\right)\text{Gr}}{1+\left(1/A_{2}\right)6\beta {\left(m\left(2\right)\right)}^{2}}, \end{array}$$

(16) $$\begin{array}{c} \theta \left(y\right)=m\left(3\right),\\ \theta^{\prime }\left(y\right)=m^{\prime }\left(3\right)=m\left(4\right),\\ \theta^{\prime \prime }\left(y\right)=m^{\prime }\left(4\right), \end{array}$$

(17) $$\begin{array}{c} m^{\prime }\left(4\right)=-\frac{1}{A}\text{PrEc}\left[{\left(1-\phi_{1}\right)}^{-2.5}{\left(1-\phi_{2}\right)}^{-2.5}{\left(m\left(4\right)\right)}^{2}+2\beta {\left(m\left(4\right)\right)}^{4}\right], \end{array}$$

and boundary conditions are

(18) $$\begin{array}{c} m_{a}\left(1\right)=0,\\ m_{a}\left(3\right)=-0.5\;\text{at }y=-1,\\ m_{b}\left(3\right)=0,\\ m_{b}\left(3\right)=0.5,\;\text{at }y=1. \end{array}$$

For the required solution, equations (14) to (18) are coded in MATLAB software.

## 5. Results and Discussion

**Figure 3:**
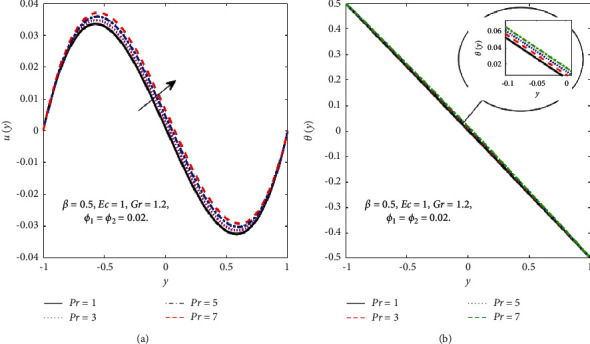
Effect of Pr on the *u*(*y*) (a) and *θ*(*y*) (b).

**Figure 4:**
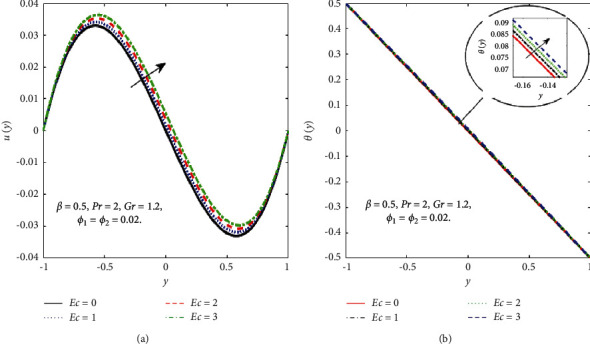
Effect of Ec on the *u*(*y*) (a) and *θ*(*y*) (b).

**Figure 5:**
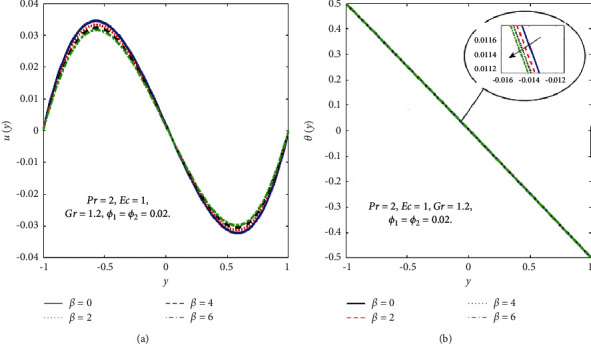
Effect of *β* on the *u*(*y*) (a) and *θ*(*y*) (b).

**Figure 6:**
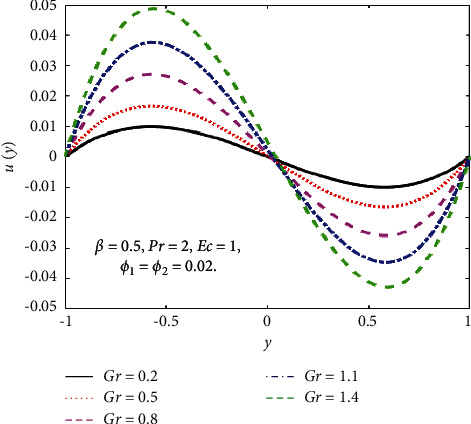
Effect of Gr on the *u*(*y*).

**Figure 7:**
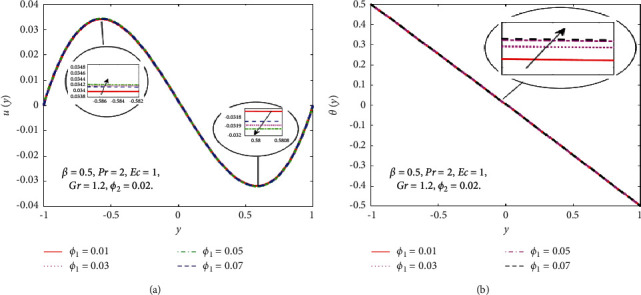
Effect of *ϕ*_1_ on the *u*(*y*) (a) and *θ*(*y*) (b).

**Figure 8:**
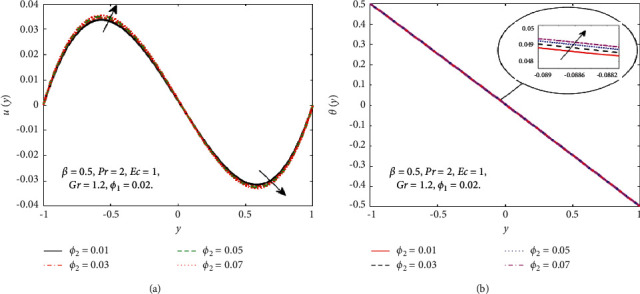
Effect of *ϕ*_2_ on the *u*(*y*) (a) and *θ*(*y*) (b).

**Figure 9:**
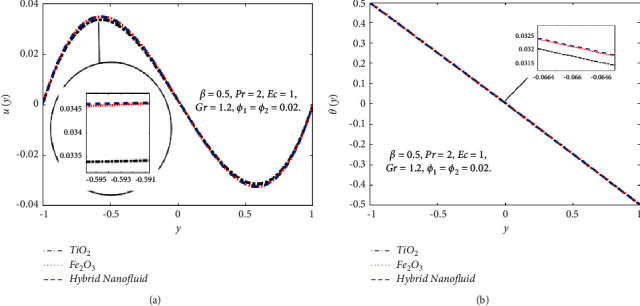
Comparison of Fe_3_O_4_, TiO_2_, and hybrid nanofluids on the *u*(*y*) (a) and *θ*(*y*) (b).

The SA is chosen as host fluid and Fe_3_O_4_+TiO_2_ are hybrid nanoparticles added into the base fluid to improve the rate of heat transfer between two vertical flat plates. The numerical solutions of governing coupled nonlinear DEs are obtained via the built-in MATLAB numerical technique bvp4c. The effect of thermo-physical parameters, such as Eckert number (Ec), Prandtl number (Pr), viscous dissipation parameter, Grashof number (Gr), third-grade fluid parameter (*β*), and nanoparticles volume fraction *ϕ*_1_ and *ϕ*_2_ on velocity and temperature fields are drawn in Figures 3–9.

Tables 3 and 4 show the comparison of velocity and temperature fields at *ϕ*_1_=*ϕ*_2_=0, *β*=0.5, Gr=Pr=*E*c=1, with studies by Ziabakhsh and Domairry [3], Manshoor et al. [5], and Biswal et al. [18, 19]. For the validation, the current study findings were found to be in excellent agreement.

Figure 3 displays the influence of the Prandtl number (Pr) on the velocity and temperature fields while other physical parameters are fixed. The velocity and temperature fields of the hybrid nanofluid rise as Pr increases due to upsurges in the thickness of the boundary layer.

The impact of the viscous dissipation parameter (Ec) on the velocity and temperature fields is demonstrated in Figure 4. It can be observed that the velocity and temperature of the hybrid nanofluid enhance with growing the values of Ec. When Ec increases, the dissipation of heat on the boundary layer region increases and also the heat transfer rate increases. The impact of third-grade fluid parameter (*β*) on the velocity and temperature field of the hybrid nanofluid is examined in Figure 5. In Figure 5(a), when *β* increases, then the velocity declines in the region −0.9 < *y* < −0.1 and it is increasing in the region 0.4 < *y* < 0.8. The reason for this is that when the viscosity of the hybrid nanofluid increases, the boundary layer thickens and the velocity declines. In Figure 5(b), when the value of *β* increases, the temperature falls. The temperature profile shows very small variations on large values of *β* because the rate of shear increases and decreases in the boundary layer thickness. The impact of buoyancy forces (Gr) on the velocity profile is portrayed in Figure 6. It can be seen that when Gr is amplified, then the velocity profile displays an increasing trend. Physically, large values of Gr boost the buoyancy force, resulting in a higher thermal force through the use of the viscous force and hence there is an upsurge in hybrid nanofluid velocity. Figures 7 and 8 demonstrate the impact of hybrid nanoparticles volume friction (*ϕ*_1_, *ϕ*_2_) on velocity and temperature fields. These profiles are plotted for hybrid nanofluids ((Fe_3_O_4_+TiO_2_)/SA). In Figure 7(a), the velocity of hybrid nanofluid increases with an increase in *ϕ*_1_ and Figure 7(b) shows the temperature profile increases because of increased heat transfer at *ϕ*_2_=0.01. The reason is that the friction of solid particles decreases the host fluid viscosity. Similarly, Figure 8(a) shows that the velocity of hybrid nanofluids increases with an increase in *ϕ*_2_ and Figure 8(b) shows that the temperature profile increases because of increased heat transfer at *ϕ*_1_=0.01. Physically, the intermolecular forces between the particles of hybrid nanofluids become weaker, and consequently, the hybrid nanofluid velocity accelerates. Further, it is detected that the thermal boundary layer thickness increases because the temperature profile increases due to higher values of hybrid nanoparticle's volume friction.  Figure 9 represents the comparison of nanofluids Fe_3_O_4_/SA and TiO_2_/SA and hybrid nanofluid ((Fe_3_O_4_+TiO_2_)/SA) at *ϕ*_1_=*ϕ*_2_=0.04. The velocity and temperature profiles of Fe_3_O_4_/SA are calculated at *ϕ*_1_=0.04 and *ϕ*_2_=0, whereas the velocity and temperature profiles of TiO_2_/SA were calculated at *ϕ*_2_=0.04 and *ϕ*_1_=0. The velocity and temperature profiles of Fe_3_O_4_ are greater than the velocity and temperature profiles of TiO_2_. Also, the velocity and temperature profiles of hybrid nanofluids ((Fe_3_O_4_+TiO_2_)/SA) are greater than Fe_3_O_4_ and TiO_2_. Physically, this is correct because Fe_3_O_4_ has a higher heat conductivity than TiO_2_. However, the temperature profile shows the same behaviour as that of the velocity profile, as Fe_3_O_4_ has larger thermal conductivity than TiO_2_. As a result, Fe_3_O_4_ conducts more heat than TiO_2_ and is less dense, resulting in Fe_3_O_4_ having a higher temperature than TiO_2_. Considering these factors, this study recommends using Fe_3_O_4_ to improve heat transmission since Fe_3_O_4_ conducts more heat and is more stable than TiO_2_.

Now, we discuss the nanoparticles volume fraction of Fe_3_O_4_(*ϕ*_1_) and TiO_2_(*ϕ*_2_) in a fuzzy environment. The nanoparticles volume fraction *ϕ*_1_ and *ϕ*_2_ are said to be TFN, as shown in Table 1, and analyzed by *α*-cut approach (0 ≤ *α* ≤ 1), as discussed in Section 3.4 in detail.

**Figure 10:**
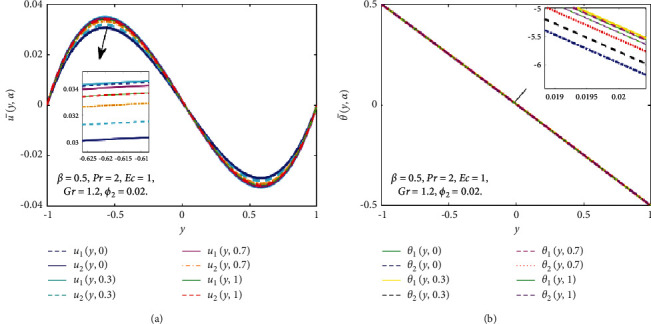
Effect of *ϕ*_1_ on the fuzzy velocity (a) and temperature (b) profiles if *ϕ*_1_ is a TFN.

**Figure 11:**
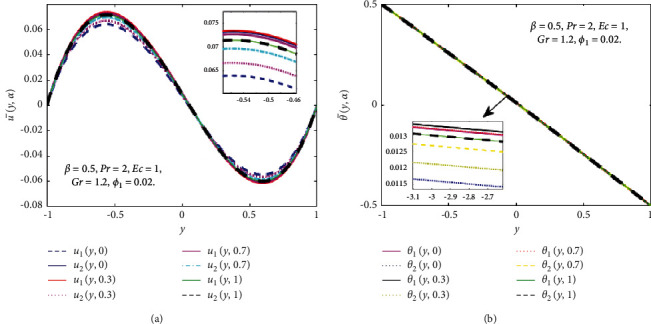
Effect of *ϕ*_2_ on the fuzzy velocity (a) and temperature (b) profiles if *ϕ*_2_ is a TFN.

Figures 10 and 11 show the nanoparticles volume fraction of Fe_3_O_4_(*ϕ*_1_) and TiO_2_(*ϕ*_2_) considered as TFNs (see in Table 2) and then the $\bar{u}\left(y,\alpha \right)$ and $\bar{\theta }\left(y,\alpha \right)$ are controlled by *α* − cut for some particular values of *α* − cut(*α*=0,0.3, 0.7, 1). In Figure 10, when *ϕ*_1_ is a TFNs, then the $\bar{u}\left(y,\alpha \right)$ and $\bar{\theta }\left(y,\alpha \right)$ convert into lower and upper bounds of the velocity and temperature fields. When *α* − cut=0, *u*_1_(*y*, *α*) and *θ*_1_(*y*, *α*) represent the nanofluid while *u*_2_(*y*, *α*)*θ*_2_(*y*, *α*) represent hybrid nanofluid at *ϕ*_2_=0.04. When *α* − cut increases the width between *u*_1_(*y*, *α*) and *u*_2_(*y*, *α*) decreases and at *α* − cut=1, they coherent with one another. It is noted that the width between *u*_1_(*y*, *α*) and *u*_2_(*y*, *α*) is very less, so the vagueness is less. Similarly, in the case of the $\bar{\theta }\left(y,\alpha \right)$, as *α* rises, the width between *θ*_1_(*y*, *α*) and *θ*_2_(*y*, *α*) reduces, and *α*=1, they are coherent with one another. It is vital to keep in mind that the width between *θ*_1_(*y*, *α*) and *θ*_2_(*y*, *α*) is quite narrow, indicating that the uncertainty is very less. Consequently, in Figure 11, when *ϕ*_2_ is a TFN, then the $\bar{u}\left(y,\alpha \right)$ and $\bar{\theta }\left(y,\alpha \right)$ convert into *u*_1_(*y*, *α*), *u*_2_(*y*, *α*), *θ*_1_(*y*, *α*), and *θ*_2_(*y*, *α*). It is essential to note that the width between the lower and upper bounds of velocity and temperature fields is very narrow, which indicates that the uncertainty is minimal.

**Figure 12:**
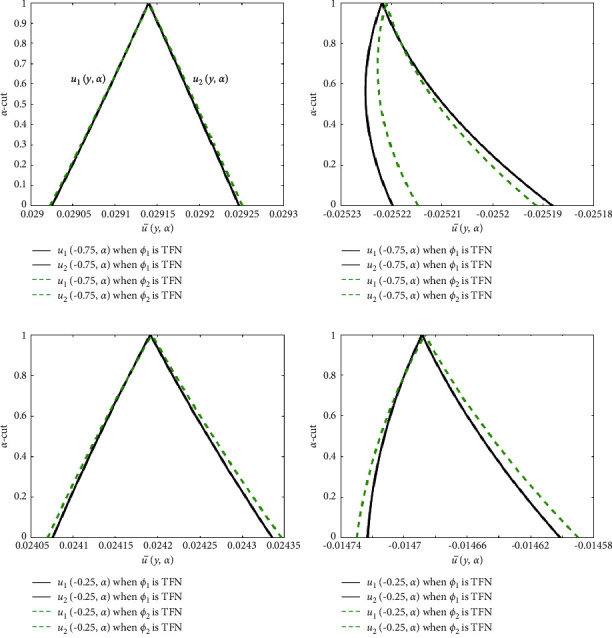
Effects of TFNs *ϕ*_2_ and *ϕ*_2_ on the fuzzy velocity profile. (a) Fuzzy velocity at *y* = −0.75. (b) Fuzzy velocity at *y* = 0.75. (c) Fuzzy velocity at *y* = −0.25. (d) Fuzzy velocity at *y* = 0.25.

**Figure 13:**
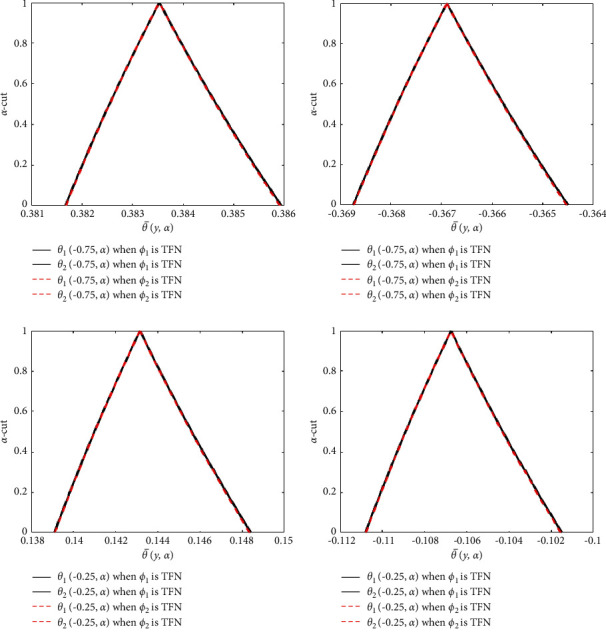
Effects of TFNs *ϕ*_2_ and *ϕ*_2_ on the fuzzy temperature profile. (a) Fuzzy temperature at *y* = −0.75. (b) Fuzzy temperature at *y* = 0.75. (c) Fuzzy temperature at *y* = −0.25. (d) Fuzzy temperature at *y* = 0.25.

Figures 12 and 13 show the triangular membership functions of the $\bar{u}\left(y,\alpha \right)$ and $\bar{\theta }\left(y,\alpha \right)$ for various values of *y*. In these diagrams, we investigated two different cases. The black lines represent the case where *ϕ*_1_ is used as the TFN and *ϕ*_2_=0.04. The green and red dashed lines indicate the representation of *ϕ*_2_ as TFN, whereas *ϕ*_1_=0.04. The horizontal axis displays the $\bar{u}\left(y,\alpha \right)$ and $\bar{\theta }\left(y,\alpha \right)$ for varying *y*, while the vertical axis displays the membership values of the $\bar{u}\left(y,\alpha \right)$ and $\bar{\theta }\left(y,\alpha \right)$ for varying *α* − cut. From Figure 12, it can be seen that the width between *u*_1_(*y*, *α*) and *u*_2_(*y*, *α*) is less, therefore the uncertainty is less for numerous values of *y*. The width between *θ*_1_(*y*, *α*) and *θ*_2_(*y*, *α*) is moderately slight in Figure 13, demonstrating that the impreciseness is neglectable for various values of *y*. As a result, the uncertain parameters are controlled through TFNs.

## 6. Conclusion

The current study focused on the natural convection flow of third-grade (Fe_3_O_4_+TiO_2_)/SA hybrid nanofluid across vertical parallel plates in a fuzzy environment. The impacts of the Eckert number (Ec), the non-Newtonian viscosity (*β*), Prandtl number (Pr), Grashof number (Gr), and nanoparticles volume fraction (*ϕ*_1_, *ϕ*_2_) on the temperature and velocity profiles have been studied for (Fe_3_O_4_+TiO_2_)/SA hybrid nanofluid. The volume fractions of nanoparticles of Fe_3_O_4_(*ϕ*_1_) and TiO_2_(*ϕ*_2_) are considered as TFNs with the help of *α* − cut (0 ≤ *α* ≤ 1) which control fuzziness. For various values of *y*, triangular membership plots of fuzzy velocity and temperature profiles were also examined. The following significant finding comes from this investigation:

  The velocity and temperature profiles rise as the values of Pr, Gr, and Ec increase, whereas the velocity and temperature profiles decrease when the value *β* increases.
  The rate of heat transfer upsurges by growing volume fractions of nanoparticles *ϕ*_1_ and *ϕ*_2_.
  The present results obtained from numerical technique via bvp4c are found to be in excellent agreement as compared to existing results.
  The hybrid nanofluid (Fe_3_O_4_+TiO_2_)/SA shows a higher heat transfer rate as compared to nanofluids Fe_3_O_4_/SA and TiO_2_/SA.
  The results indicate that the crisp solution is always in-between the upper and lower solutions when *α* − cut to increase from 0 to 1.
  The sensitivity of the assumed TFN is held influenced by the unfluctuating width of the fuzzy velocity or temperature.
  According to the triangular membership plots, the uncertain width of the fuzzy velocity and temperature is less, so the assumed TFNs are less sensitive. Finally, the TFN is represented visually for better understanding. As a result, the TFNs may be used to different heat transfer problems.

## Figures and Tables

**Table 1:** Thermo-physical properties of base fluids and hybrid nanoparticles [14, 20].

Materials	*ρ* (kg/m^3^)	*Cρ*(J/kg^−1^k^−1^)	*K* (W/m)	*β*_*T*_ (k^−1^)
Sodium alginate (SA)	989.0	4175.0	0.6376.0	99.0
Ferro-particle (Fe_3_O_4_)	5180.0	670.0	9.70	1.18 × 10^−5^
Titanium dioxide (TiO_2_)	4250.0	686.20	8.95380	0.9 × 10^5^

**Table 2:** TFNs of fuzzy nanoparticles of volume fraction.

Fuzzy number	Crisp value	TFN	*α* − cut approach
*ϕ*_1_	[0.01–0.04]	[0, 0.05, 0.1]	[0.05*α*, 0.1 − 0.05*α*], *α* ∈ [0,1]
*ϕ*_2_	[0.01–0.04]	[0, 0.05, 0.1]	[0.05*α*, 0.1 − 0.05*α*], *α* ∈ [0,1]

**Table 3:** Comparison of velocity profile when *ϕ*_1_=*ϕ*_2_=0, *β*=0.5, Gr=Pr=*E*c=1, with the existing result for regular fluid.

*Y*	Present results (bvp4c)	Ziabakhsh and Domairry [3] (HAM)	Biswal et al. [18] (HPM)	Biswal et al. [19] (GM)	Manshoor et al. [5] (VPM)
−1	0	0	0	0	0
−0.8	0.02244430	0.02391937	0.02416171	0.02368610	0.033923604
−0.6	0.03122643	0.03217274	0.03262933	0.03170120	0.032183540
−0.4	0.02712964	0.02840695	0.02901579	0.02794809	0.027143138
−0.2	0.01603016	0.01661778	0.01731154	0.01632954	0.016274634
0	−0.00002592	0.00080780	0.00152009	0.00074834	0.000922405
0.2	−0.01448865	−0.01508225	−0.01441546	−0.01489272	−0.015143973
0.4	−0.02683483	−0.02710348	−0.026554082	−0.02669087	−0.028257013
0.6	−0.03070475	−0.03122988	−0.03081889	−0.03074332	−0.031223835
0.8	−0.02313980	−0.02342875	−0.02320304	−0.02314729	−0.023274354
1	0	0	0	0	0

**Table 4:** Comparison of temperature profile when *ϕ*_1_=*ϕ*_2_=0, *β*=0.5, Gr=Pr=*E*c=1, with the existing result for regular fluid.

*Y*	Present results (bvp4c)	Ziabakhsh and Domairry [3] (HAM)	Biswal et al. [18] (HPM)	Biswal et al. [19] (GM)	Manshoor et al. [5] (VPM)
−1	0.49999999	0.49999999	0.49987599	0.50000000	0.500000000
−0.8	0.40009178	0.40073588	0.40157624	0.40097357	0.400246306
−0.6	0.30116719	0.30117737	0.30269966	0.30172607	0.309367078
−0.4	0.20863343	0.20159090	0.20321740	0.20225927	0.201548465
−0.2	0.10108217	0.10192749	0.10350286	0.10257493	0.101925345
0	−0.00299024	0.00206049	0.00350177	0.00267484	0.002174325
0.2	−0.09501960	−0.09807006	−0.09677903	−0.09743924	−0.098174536
0.4	−0.19536608	−0.19840851	−0.19733286	−0.19776553	−0.198546725
0.6	−0.29679969	−0.29882852	−0.29812783	−0.29830227	−0.298765434
0.8	−0.39825954	−0.39927474	−0.39909138	−0.39904768	−0.400465233
1	−0.49999999	−0.500000000	−0.50012401	−0.50000000	−0.500000000

## Data Availability

No data were used in this article.
